# The Assessment of Calcium and Bleomycin Cytotoxic Efficiency in Relation to Cavitation Dosimetry

**DOI:** 10.3390/pharmaceutics15051463

**Published:** 2023-05-11

**Authors:** Martynas Maciulevičius, Renaldas Raišutis, Baltramiejus Jakštys, Linas Svilainis, Andrius Chaziachmetovas, Saulius Šatkauskas

**Affiliations:** 1 Biophysical Research Group, Faculty of Natural Sciences, Vytautas Magnus University, Vileikos St. 8, LT-44404 Kaunas, Lithuania; baltramiejus.jakstys@vdu.lt (B.J.); saulius.satkauskas@vdu.lt (S.Š.); 2 Ultrasound Research Institute, Kaunas University of Technology, K. Baršausko St. 59, LT-51423 Kaunas, Lithuania; renaldas.raisutis@ktu.lt; 3 Department of Electrical Power Systems, Faculty of Electrical and Electronics Engineering, Kaunas University of Technology, Studentų St. 48, LT-51367 Kaunas, Lithuania; 4 Electronics Engineering Department, Kaunas University of Technology, LT-51368 Kaunas, Lithuania; linas.svilainis@ktu.lt (L.S.); andrius.chaziachmetovas@ktu.lt (A.C.)

**Keywords:** calcium, bleomycin, microbubble, cavitation, ultrasound, sonoporation

## Abstract

Microbubble (MB)- and ultrasound (US)-facilitated intracellular Ca^2+^ delivery, known as sonoporation (SP), is a promising anticancer treatment modality, since it allows a spatio-temporally controllable and side-effect-free alternative to conventional chemotherapy. The current study provides extensive evidence that a 5 mM concentration of Ca^2+^ in combination with US alone or US and Sonovue MBs can be an alternative to the conventional 20 nM concentration of the anticancer drug bleomycin (BLM). Ca^2+^ application together with SP induces a similar level of death in Chinese hamster ovary cells to the combination of BLM and SP but does not cause systemic toxicity, as is inherent to conventional anticancer drugs. In addition, Ca^2+^ delivery via SP alters three vital characteristics essential for viable cells: membrane permeability, metabolic activity and proliferation ability. Most importantly, Ca^2+^ delivery via SP elicits sudden cell death—occurring within 15 min—which remains similar during 24–72 h and 6 d periods. The extensive study of US waves side-scattered by MBs led to the quantification of the cavitation dose (CD) separately for subharmonics, ultraharmonics, harmonics and broadband noise (up to 4 MHz). The CD was suitable for the prognostication of the cytotoxic efficiency of both anticancer agents, Ca^2+^ and BLM, as was indicated by an overall high (R^2^ ≥ 0.8) correlation (22 pairs in total). These extensive analytical data imply that a broad range of frequencies are applicable for the feedback-loop control of the process of US-mediated Ca^2+^ or BLM delivery, successively leading to the eventual standardization of the protocols for the sonotransfer of anticancer agents as well as the establishment of a universal cavitation dosimetry model.

## 1. Introduction

Sonoporation (SP) involves the non-invasive application of therapeutic ultrasound (US) in conjunction with US contrast agents known as microbubbles (MBs), with the aim to deliver exogenous bioactive compounds, including anticancer agents and nucleic acids, to cells and tissues [1,2,3,4]. It is a promising method for therapeutic applications, since US-MB-enhanced molecular delivery is a non-invasive technique that is safe—as it poses no risks associated with eliciting the immune response—as well as cost-effective and available for wide-ranging use. The employment of MBs as biological vectors is considered to be superior to chemical and viral adjuvants due to low immunogenicity and mutagenicity. MBs bearing liposomes loaded with therapeutics and MBs, targeted via ligand–receptor interactions, are exploited as carriers of anticancer drugs, which increase the efficiency of payload delivery to site-specific locations with minimal off-target delivery [4,5,6].

The successful application of SP to fight cancer has been shown in a plethora of research and is conducted with the aim to facilitate the death of cancerous cells, as well as enhance the regression of malignant tumors. Escoffre et al. reported the efficient reduction in the viability of glioblastoma (U-87MG) and breast cancer (MDA-MB-231) cells down to 20 and 47%, respectively, after doxorubicin (DOX) delivery via US-MB-enhanced treatment [1]. Lentacker et al. exploited upgraded MBs bearing DOX-loaded liposomes and successfully applied them in therapy against melanoma cells [5]. The researchers reported two-to-six-fold higher efficiency as compared to a liposome and US combination. Tamosiunas et al. showed that cell death, elicited by bleomycin (BLM), was enhanced as the MB sonodestruction rate was increased [7]. The cytolethal effect of BLM was optimized by adjusting the exposure duration corresponding to the duration necessary to acquire complete MB sonodestruction [8]. In trials with animals, Sonoda et al. showed that the simultaneous employment of BLM together with SP resulted in a significant reduction in tumor weight 4 d after treatment as compared to the control group [2]. Iwanaga et al. almost completely eradicated pernicious squamous carcinoma tumors after a 4-week treatment using a low dose of BLM delivered via SP in mice [9]. Finally, the contemporaneous employment of the anticancer drug gemcitabine in conjunction with MBs and US irradiation has led to encouraging results in clinical practice for therapy against malignant pancreatic tumors [10]. However, conventionally exploited chemotherapeutics in the field, e.g., BLM [2,7,8,9] and DOX [1,5,11], are cytolethal and, therefore, cause systemic toxicity to live organisms. The detrimental secondary effects of BLM are nausea and/or vomiting, commonly accompanied by pulmonary toxicity in some cases, leading to irreversible, harmful disease, as well as significantly reduced bioavailability [12]. The collateral aftermath of DOX is reported to be bone marrow depression, cardiomyopathy leading to heart failure, general nephrotoxicity and glomerular atrophy [13].

Calcium ion delivery, enhanced by different intracellular delivery modalities, such as electroporation (EP) [14,15] and SP [16], has been proposed as a favorable alternative to regular chemotherapy. A plethora of extracellular stimuli are able to implicitly increase the intracellular level of diffusible Ca^2+^, which, in turn, acts as a second messenger and controls a milieu of intrinsic cellular processes, including the mechanism of cell death. An increase in Ca^2+^ levels in live organisms is effectively mediated by the homeostatic systems within the cell. Therefore, in comparison to traditional chemotherapy, the employment of US-MB-mediated delivery of supra-physiological Ca^2+^ levels for cancer treatment can significantly diminish the serious after-effects inflicted by conventional cytotoxic drugs, particularly following intravascular or oral administration. However, Ca^2+^ delivery and possible therapeutic applications are poorly investigated in the field of SP, except for our previous work [16]. Therefore, in the first part of our research, we aimed to determine the possible Ca^2+^ applications for chemotherapy by comparing the levels of cell death induced in Chinese hamster ovary (CHO) cells by both Ca^2+^ and BLM after SP.

The efficacy of US-mediated delivery is highly increased if therapeutic agents are delivered together with MBs—inert gas nuclei embedded within a biocompatible shell [4,8,17]. During their exposure to an external US field, MBs undergo cavitation—MB oscillation (stable cavitation)—followed by subsequent implosion (inertial cavitation), which creates liquid microstreams and microjets that are able to induce the transient permeabilization of the cell membrane by creating pores and/or facilitating endocytotic processes [3,18,19,20]. MBs, constantly oscillating in the regime of stable cavitation, generate microstreams—circular medium flows that lead to shear forces—and, consequently, the infliction of shear stress on the surface of the cell membrane. On the contrary, the implosion of MBs results in violent phenomena, such as microjets or shock waves [21,22].

Since US-MB interplay is followed by the collapse of cavitation nuclei, which inflict irremediable membrane wounds that eventually lead to cell death, the intracellular sonotransfer of bioactive compounds is followed by a considerable degree of cell death. Thus, the level of inertial cavitation must be strictly monitored throughout the procedure of SP. With the aim to sustain the most promising feature of cell SP—the spatio-temporal control of the delivery of cytolethal substances—the establishment of a standardized and accurate cavitation dosimetry model is crucial. This introduces an option to reduce the intense cell-injury-associated secondary aftermath of inertial cavitation and, in tandem, ensure the attainment of the required efficiency of the transfer of anticancer agents.

The experimental results on anticancer drug delivery efficiency considerably vary among different studies performed in the field [1,7,17,23,24,25]. The plethora of variable biophysicochemical parameters (setups, US parameters, media, MB type, cell type, etc.) exploited by different researchers in the field accounts for the diversity of attained data [11,25,26]. Moreover, these “ingoing” experimental conditions only implicitly relate to the extent of US-evoked MB cavitation and the level of biological effect. Furthermore, considering the complexity and activation of the immune response of a live organism, directly extrapolating optimized in vitro settings to in vivo practice becomes problematic. This drawback of direct in vitro–in vivo translation hinders progress and requires restarting the optimization process from scratch for animal trials. The optimization of biophysical conditions and the eventual development of a standardized in vivo protocol for SP are among the key points of SP research in general.

Ever since it was evidenced that the regime of US-elicited MB cavitation is able to facilitate the inward flux of exogenous therapeutic agents, an open-ended debate concerning the prevalent cavitation pattern has been ongoing in the scientific community [27]. By passively registering scattered US signals originating from cavitating MBs and analyzing their contents, it is possible to monitor MB behavior. During stable cavitation, MBs oscillate periodically and linearly in response to the changes in the external US field. Therefore, the frequency spectrum associated with stable cavitation contains distinct harmonic (2*F*, 3*F*, 4*F*…), subharmonic (*F*/2, *F*/3, *F/*4…) and ultraharmonic (3*F*/2, 5*F*/2, 7*F/*2…) constituents of the center frequency (*F*) [28,29]. Once exposed to higher pressures in the acoustic field, MBs exhibit rapid volumetric expansion, followed by successive collapse. Therefore, the spectrum attributed to inertial cavitation bears the characteristic appearance of frequency constituents extensively distributed throughout the whole range of frequencies. Thus, in the current research, when performing cavitation dosimetry, we focused on the frequency regions associated with harmonics, subharmonics, ultraharmonics and broadband noise, with the possibility of facilitating the discussion on the prevalent regime of cavitation.

## 2. Materials and Methods

### 2.1. Cell Culture

Chinese hamster ovary (CHO) cells were kindly provided by Dr. P. Boukany (Delft University of Technology, Delft, The Netherlands). Cells were cultured in Dulbecco’s Modified Eagle Medium (DMEM) (Sigma Aldrich, St. Louis, MO, USA) supplemented with 10% non-heat-inactivated fetal bovine serum (FBS) (Sigma Aldrich, USA) as well as solutions of 1% L-glutamine (Invitrogen Inc., Carlsbad, CA, USA) and 100 U/mL penicillin with 100 μg/mL streptomycin (Sigma Aldrich, USA). Cells were cultured in single layers in 10 cm diameter Petri dishes (TPP, Trasadingen, Switzerland) at a 37 °C temperature in a 5% CO_2_ atmosphere. Trypsin-EDTA solution (Sigma Aldrich, USA) was used for the harvesting of the cells.

### 2.2. Experimental System

**Figure 1:**
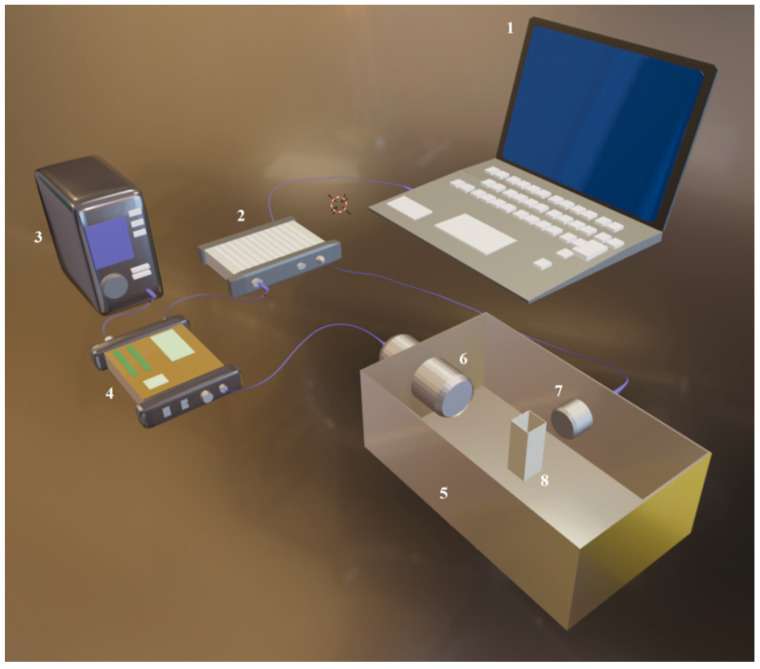
Experimental system used for SP experiments. 1—personal computer; 2—waveform generator/oscilloscope; 3—high-voltage power supply; 4—push–pull topology pulser; 5—experimental bath; 6—excitation transducer; 7—receiver, 8—cuvette.

Experiments were conducted using an experimental system (Figure 1) composed of a plastic chamber supplied with degassed water; US transducers; and hardware designed for the registration of US signals [11].

US signals were generated and registered by an arbitrary signal generator/oscilloscope (Picoscope 5242B, Picotech, Eaton Socon, UK). A push–pull transformer (SE-TX02-00, Kaunas University of Technology, Kaunas, Lithuania) [30,31], powered by a high-voltage supply (MCP Lab Electronics, Shanghai, China), was utilized for the amplification of electrical signals. US pulses were generated by an 18 mm diameter unfocused transducer (0.9–1.2 MHz at −6 dB bandwidth) operating at a center frequency of 1 MHz (Medelkom, Vilnius, Lithuania). A hydrophone (HNR 1000, Onda Corp., Sunnyvale, CA, USA) was utilized to obtain US peak negative acoustic pressure within the experimental cuvette. The interior of the experimental chamber was lined with an acoustic absorber (AptFlex F28, Precision Acoustics, Dorchester, UK) with the aim to reduce the reflections of US waves. Experiments were conducted at room temperature (24 °C) in a 1 cm wide SP cuvette (Plastibrand, Bonn, Germany), administered with a 1 mL volume.

For the passive detection of the process of cavitation, an 8 mm diameter transducer with a center frequency of 5 MHz (2.1–7.9 MHz at bandwidth of −6 dB) (Doppler Electronic Technologies, Guangzhou, China) was utilized and placed perpendicularly to the excitation transducer.

### 2.3. Experimental Procedure

A CHO cell suspension containing CaCl_2_ (Lachema, Brno, Czech Republic) or bleomycin (BLM) (Teva, Amsterdam, The Netherlands) was exposed to US with or without the administration of Sonovue MBs (Bracco diagnostics Inc., Cadempino, Switzerland). Sonovue MBs were prepared in 0.9% NaCl solution, as indicated in the instruction sheet provided by the manufacturer. The experiments were performed in 0.9% NaCl solution administered with CaCl_2_ at varying concentrations (0–20 mM) or BLM at a constant concentration (20 nM). The concentrations of MBs and cells were (2 ± 0.1) × 10^7^ MBs/mL and 0.9 × 10^6^ cells/mL, respectively. To guarantee the similarity of the samples, the concentration of MBs was assessed for each sample using an optical technique as reported previously [11].

The experiments were classified into the following series:

Ca^2+^ series. The experimental groups for US alone were (1) the control group (no treatment); (2) the US group (US); (3) the group treated with Ca^2+^ in combination with US (Ca^2+^ + US). The experimental groups for the US and MB combination were (1) the control group (no treatment); (2) the cavitation group (MB + US); (3) the therapeutic group (Ca^2+^ + MB + US).

BLM series. The experimental groups for US alone were (1) the control group (no treatment); (2) the US group (US); (3) the group treated with BLM in combination with US (BLM + US). The experimental groups for the US and MB combination were (1) the control group (no treatment); (2) the cavitation group (MB + US); (3) the therapeutic group (BLM + MB + US).

**Figure 2:**
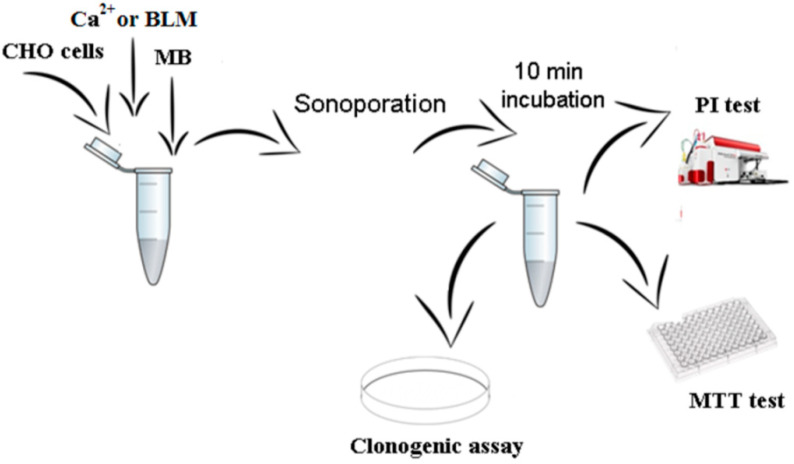
A demonstration of the procedure of SP experiments. CHO cells were subjected to SP treatment using either Ca^2+^ or BLM, followed by 10 min incubation. Subsequently, separate assays (PI, MTT and clonogenic) for the evaluation of cell viability were performed.

The procedure of the experiment is demonstrated in Figure 2.

US at 1 MHz center frequency, 1 kHz pulse repetition frequency, 10% duty cycle (100 µs on, 900 µs off) and 0–700 kPa peak negative acoustic pressure was exploited for the irradiation of cells, with an exposure duration of 6 s.

After exposure to US, cells were incubated for 10 min at a 37 °C temperature in a 5% CO_2_ atmosphere. Subsequently, they were resuspended in the growth medium for the following evaluation of the viability of cells using the assays listed below.

### 2.4. Cell Viability Evaluation

#### 2.4.1. Propidium Iodide Assay

The propidium iodide (PI) assay is a cell viability test based on the assessment of the integrity of the cell membrane. The flux of PI to the interior of the cell occurs due to the deficit or complete loss of the barrier function of the cell membrane. After the incubation of cells, the medium was removed from the samples of cells by centrifugation. Cells were then resuspended in 1× PBS (Lonza Inc., Morristown, NJ, USA) and administered PI (40 µM final concentration). The percentage of PI-positive cells was estimated by flow cytometry (BD Accuri C6, Accuri Cytometers Inc., Ann Arbor, MI, USA) at progressive time points after SP: 15 min, 1 h, 2 h, 3 h and 5 h.

For the visualization of PI in the interior of the cell, PI was administered to the suspension of cells 15 min after SP, and 40 µL was transferred onto the objective glass and covered with a 22 × 22 mm glass cover slip (Carl Roth, Karlsruhe, Germany). After additional incubation for 5 min (required for the cells to attach to the surface of the objective glass), the cells were photographed. Bright-field and fluorescent images of the same cell were taken successively. The imaging of the cells was performed with a fluorescence microscope (Motic AE31, Motic, Richmond, BC, Canada) equipped with a camera (MoticamPro, Motic, Canada) and filter cube (D560/40X excitation, dichroic 595DCLP mirror, D630/60 emission). The photographs were obtained using Motic Image Advanced 3.2 software.

#### 2.4.2. MTT Assay

The MTT assay is a colorimetric test developed for the evaluation of the viability of cells according to the state of their metabolic function. Dead/damaged cells lose their enzymatic activity and become incapable of producing a conformational change in MTT salt. After incubation, cells were cultured in a 96-well microplate (Plastibrand, Germany) supplemented with growth medium for 24, 48 and 72 h. Two hours before measurements were taken, the cells were administered fresh growth medium and a solution of MTT salt (0.5 mg/mL final concentration), followed by incubation. Subsequently, the growth medium was disposed of, and the wells of the microplate were washed with 1× PBS. The formazan salt produced within the cells was dissolved in isopropanol (Chempur, Piekary Śląskie, Poland). For the measurement of optical absorption, the content of each well was transferred into the respective well of another transparent 96-well microplate. The optical density of the suspension was measured at a 550 nm wavelength using a microplate reader (Spectrostar Nano, BMG Labtech, Ortenberg, Germany); the obtained values of absorption were corrected by subtracting the background and were normalized to the values of the control samples (no treatment).

#### 2.4.3. Cell Clonogenic Assay

The cell clonogenic assay (CA) is a test designed to determine the viability of cells by evaluating the ability of live cells to proliferate. Dead/damaged cells are unable to divide and, therefore, unable to produce replicas of themselves and form colonies. After the incubation of cells, ~330 cells were transferred to 4.1 cm^2^ tissue culture dishes (TPP, Switzerland) supplemented with 2 mL of growth medium. The cells were cultured for 6 days and then fixed using 1 mL of 96% ethanol for 10 min. Subsequently, colonies of cells were stained using crystal violet solution (Sigma Aldrich, USA) (containing 2.3% crystal violet, 0.1% ammonium oxalate and 20% ethanol). The number of formed colonies was evaluated using an optical microscope (MBS 9, LOMO, St. Petersburg, Russia) and normalized to the control group (no treatment). Since the results of cell viability/death obtained after 6 d using clonogenic assay represent the final viability or death of cells [32], the results are termed “cell viability” or “cell death”. Conversely, the data obtained using PI and MTT assays are termed “permeabilization” and “metabolic activity”, respectively.

### 2.5. The Quantification of the Cytotoxic Efficiency of Anticancer Agents

BLM or Ca^2+^ delivery efficiency in the (BLM + MB + US) or (Ca^2+^ + MB + US) group may be an outcome of (i) cell death induced by exposure to US alone and/or US-elicited MB cavitation (MB + US); (ii) cells killed by BLM alone, MBs or their combined application (BLM + MB) for the BLM series, or similarly, Ca^2+^ alone, MBs or their combined application (Ca^2+^ + MB) for the Ca^2+^ series; (iii) intracellular BLM or Ca^2+^ transfer to reversibly permeabilized cells, resulting in the death of cells caused by the intracellular toxicity of BLM or Ca^2+^. Therefore, with the aim to determine the efficiency of BLM or Ca^2+^ delivery, the percentages of cell death attained with the cell clonogenic assay in the (MB + US) and (BLM + MB) or (Ca^2+^ + MB) groups were subtracted from the corresponding percentage achieved in the (BLM + MB + US) or (Ca^2+^ + MB + US) group, respectively, depending on whether the cells were exposed to BLM or Ca^2+^. In the figures, the percentage for the (BLM + MB) or (Ca^2+^ + MB) group is located on the curve of (BLM + MB + US) or (Ca^2+^ + MB + US), respectively, at 0 kPa acoustic pressure. Similarly, in order to determine the efficiency of BLM or Ca^2+^ delivery induced by exposure to US alone (in the absence of MBs), the subtraction of the percentages of cell death (obtained using the cell clonogenic assay) was completed for BLM using the equation (BLM + US) − (US) − (BLM); for Ca^2+^, the calculation was (Ca^2+^ + US) − (US) − (Ca^2+^).

The results provided by the cell clonogenic assay are presented as cell death in Figure 6, since the final outcome, the cytotoxicity of the therapeutic agent, is quantified as the difference in cell death in particular experimental groups. Conversely, in Figure 11, the results are presented as cell viability, since the main outcome, beneficial for the reader, would be the ability to control (preserve) a particular level of cell viability during the treatment. The relation between cell viability and cell death is simply defined as 100% − cell viability = cell death.

### 2.6. Signal Analysis

#### 2.6.1. Signal Registration

MB cavitation signals were recorded using a passive cavitation detection (PCD) system. PCD was performed using a passively coupled transducer perpendicularly aligned to the excitation transducer for the acquisition of the signals. The signal for an overall 6 s duration was recorded at a 31.25 MS/s sampling rate and 8-bit hardware resolution in 100 frames (16.7 frames/s); the duration of every frame was 10 ms (includes 10 recorded pulses).

The signals of US scattering were registered for two instances: (i) in the presence of MBs (+MB group); (ii) in the absence of MBs (−MB group/background group).

#### 2.6.2. Signal Processing

**Figure 3:**
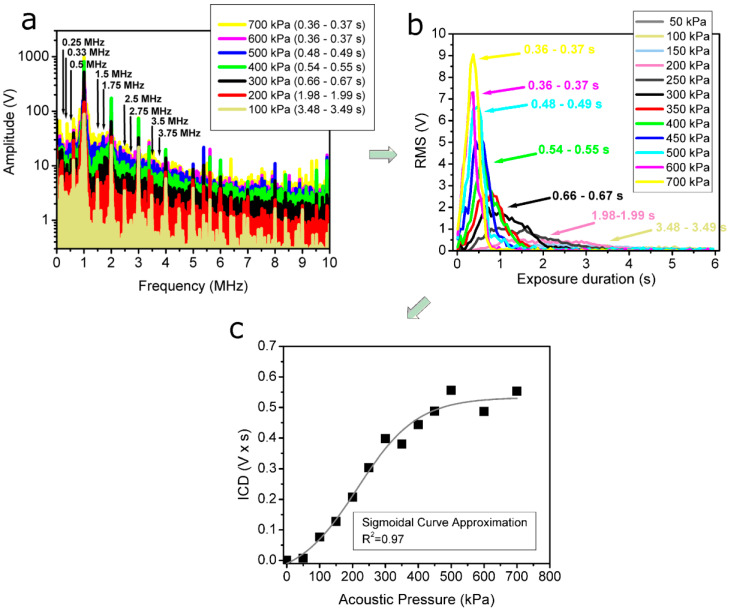
FFT of the acoustic pulses at different acoustic pressures (**a**), where arrows indicate specific harmonic, subharmonic and ultraharmonic components of the fundamental frequency selected for RMS quantification; differential RMS at different acoustic pressures, quantified in the frequency band of 1.4–1.6 MHz (around the first ultraharmonic—1.5 MHz) of the FFT spectrum (**b**); CD, calculated as the integral of differential RMS temporal curves, obtained at different acoustic pressures (**c**).

The frequency spectra of acoustic signals were determined using the fast Fourier transform (FFT). Figure 3a presents the frequency spectra of acoustic signals registered in the frames, corresponding to the highest RMS values at specific acoustic pressures displayed in Figure 3b. The frequency spectra indicate the highest amplitudes of as well as pronounced changes in harmonic, subharmonic and ultraharmonic constituents of the frequencies occurring in the lower-frequency range (up to around 4 MHz). Correspondingly, the frequency components (indicated by arrows in Figure 3a) associated with specific harmonics, subharmonics and ultraharmonics of the excitation frequency were selected for subsequent analysis and RMS quantification (Figure 3b).

#### 2.6.3. Estimate Quantification

Root mean square (RMS) values were quantified for US signals registered in the presence and absence of MBs in different ranges of frequency within the FFT spectrum using Equation (1):

(1) $$\mathrm{RMS}=\sqrt{\frac{1}{n}\left(x_{1}^{2}+x_{2}^{2}+\cdots +x_{n}^{2}\right)\;}$$

where *n* is the number of components in the frequency spectrum, acquired after FFT; *x* is the amplitude value of the component corresponding to a specific value of the frequency.

Frequency ranges for RMS and CD calculations were selected to be ± 0.1 MHz around (i) subharmonics (1st = 0.5 MHz, 2nd = 0.33 MHz and 3rd = 0.25 MHz), (ii) harmonics (2nd = 2 MHz and 3rd = 3 MHz), (iii) ultraharmonics (1st = 1.5 MHz, 2nd = 2.5 MHz and 3rd = 3.5 MHz) and (iv) broadband noise (1.75, 2.75 and 3.75 MHz—arbitrary selected frequency range not overlapping with subharmonics, ultraharmonics or harmonics). Thus, the respective frequency ranges are as follows: (i) subharmonic (0.4–0.6, 0.33–0.43 and 0.15–0.35 MHz), (ii) harmonic (1.9–2.1 and 2.9–3.1 MHz), (iii) ultraharmonic (1.4–1.6, 2.4–2.6 and 3.4–3.6 MHz) and (iv) broadband noise (1.65–1.85, 2.65–2.85 and 3.65–3.85 MHz). RMS quantified in the harmonic 0.9–1.1 MHz frequency range unsurprisingly resulted in an attenuation-like curve of the frequency of excitation; therefore, this curve was not used for the analysis, as it does not correspond to US scattering. Differential RMS was quantified by subtracting the RMS of the −MB group (RMS_−MB_) from the RMS of the +MB group (RMS_+MB_) with the aim to reduce the influence of systemic background on the results:

(2) $$\mathrm{Differential}\;\mathrm{RMS}\left(t\right)={\left(\mathrm{RMS}\right)}_{+\mathrm{MB}}\;\left(t\right)-{\mathrm{RMS}}_{-\mathrm{MB}\;}\left(t\right)$$

where *t* is exposure duration (or time).

The curves of differential RMS quantified in the 1.4–1.6 MHz frequency range (around the first ultraharmonic—1.5 MHz) at different acoustic pressures are given as an example in Figure 3b. In order to obtain the differential cavitation dose (CD), or simply CD, differential RMS was integrated with respect to the exposure duration [26].

(3) $$CD=\int_{0}^{t_{F}}\mathrm{Differential}\;\mathrm{RMS}\;\left(t\right)\;dt,$$

where *CD* is the cavitation dose, *t* is the exposure duration (or time), 0 specifies 0 s (the start of US application), and *t_F_* signifies the final exposure duration (time at the end of integration).

The *CD* curve calculated in the frequency range of 1.4–1.6 MHz, and accurately approximated with sigmoidal function, is given as an example in Figure 3c.

For the spectral analysis (presented in Figure 9b), RMS was quantified at ±0.1 MHz around (i) subharmonics (1st = 0.5 MHz, 2nd = 0.33 MHz, 3rd = 0.25 MHz, 4th = 0.2 MHz, 7th = 0.125 MHz and 9th = 0.1 MHz), (ii) harmonics (2nd = 2 MHz, 3rd = 3 MHz, 4th = 4 MHz, 5th = 5MHz, 7th = 7 MHz and 9th = 9 MHz), (iii) ultraharmonics (1st = 1.5 MHz, 2nd = 2.5 MHz, 3rd = 3.5 MHz, 4th = 4.5 MHz, 5th = 5.5 MHz, 7th = 7.5 MHz and 9th = 9 MHz) and (iv) broadband noise (1.75, 2.75, 3.75, 4.75, 5.75, 7.75 and 9.75 MHz). Thus, the respective frequency ranges were as follows: (i) subharmonic (0.4–0.6, 0.33–0.43, 0.15–0.35, 0.1–0.3, 0.025–0.225 and 0–0.2 MHz), (ii) harmonic (1.9–2.1, 2.9–3.1, 3.9–4.1, 4.9–5.1, 6.9–7.1 and 8.9–9.1 MHz), (iii) ultraharmonic (1.4–1.6, 2.4–2.6, 3.4–3.6, 4.4–4.6, 5.4–5.6, 7.4–7.6 and 9.4–9.6 MHz) and (iv) broadband noise (1.65–1.85, 2.65–2.85, 3.65–3.85, 4.65–4.85, 5.65–5.85, 7.65–7.85 and 9.65–9.85 MHz).

### 2.7. Statistical Analysis

Data in the Results section are presented as the mean ± standard error of the mean (SEM). The mean ± SEM for each experimental point was calculated from at least 4 experimental replicates. The Mann–Whitney test was performed in order to determine statistical significance between two groups. The statistical significance for multiple comparisons was evaluated using one-way ANOVA. The dependence between the results of SP and CD was estimated using correlation analysis; the coefficient of correlation determination (R^2^) is reported to define the strength of the correlation. Matlab (Mathworks, Natick, MA, USA) and Origin (OriginLab Co., Wellesley Hills, MA, USA) software were used for the analysis of the data.

## 3. Results

### 3.1. The Influence of Intracellular Ca^2+^ Delivery for Cell Permeabilization and Cell Viability at Constant Acoustic Pressure

**Figure 4:**
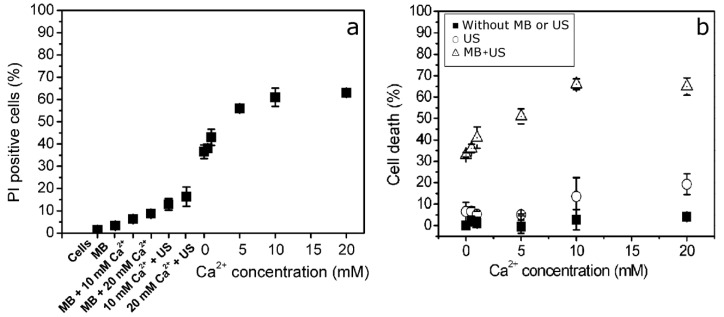
Cell membrane permeability evaluated for different treatment groups using PI assay, performed 15 min after SP (**a**); cell viability evaluated for different treatment groups using clonogenic assay, performed 6 d after SP (**b**).

We evaluated the impact of different Ca^2+^ concentrations on the integrity of the cell membrane. For this purpose, Ca^2+^ together with MBs was administered before US (300 kPa) application, while the cell membrane permeability marker, PI, was administered after SP. The obtained results indicate that an increase in Ca^2+^ concentration facilitates an inward PI flux in up to ~65% of cells from the total cell population (Figure 4a).

The progressive loss of cell membrane barrier function increases with up to a 5 mM Ca^2+^ concentration and then enters the plateau phase. The results also indicate that the administration of MBs and Ca^2+^ without US exposure, as well as the administration of Ca^2+^ alone followed by subsequent US treatment, had a low impact on cell membrane permeability. Therefore, US-elicited MB cavitation is a requisite for Ca ions to alter the integrity of the membranes of CHO cells.

In the next step, we evaluated the impact of Ca^2+^ on cell viability by evaluating the ability of cells to proliferate, since cell proliferation is a key factor for cells to be indicated as “viable”. Therefore, the impact of Ca ions on the efficiency of cell colony formation was evaluated in the absence and presence of US (300 kPa) exposure, as well as after combined cell treatment using MBs and US (300 kPa). The obtained results (Figure 4b) indicate that an increase in Ca^2+^ concentration without US exposure had an insignificant effect on cell proliferation; similarly, Ca^2+^ addition to the medium followed by US irradiation did not induce additional cell death as compared to the control (0 mM Ca^2+^ concentration), except for a minimal decrease in cell viability observed at the highest (20 mM) Ca^2+^ concentration.

After the exposure of cells to simultaneous MB and US treatment, we observed a gradual decrease in cell viability from 0.5 to 1 mM Ca^2+^ concentrations, while the viability decrease entered a plateau phase at a 5 mM Ca^2+^ concentration (Figure 4b). This finding is in accordance with the results of cell permeabilization analyses (Figure 4a). Since cell viability did not significantly decrease as Ca^2+^ was increased to 10 mM or 20 mM, the concentration of 5 mM was selected for further experiments.

**Figure 5:**
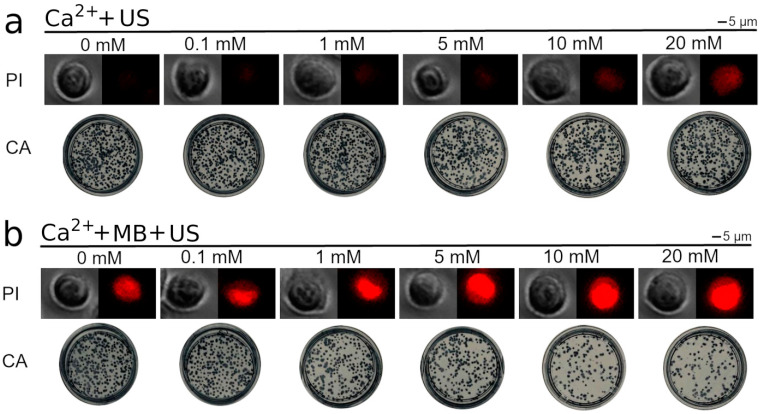
Cell membrane permeability (PI assay, PI) and cell viability (clonogenic assay, CA) depending on different Ca^2+^ concentrations after US (**a**) or US + MB (**b**) treatment at 300 kPa acoustic pressure.

The images corresponding to the results presented in Figure 4 and illustrating a successive PI increase inside the cell, as well as a progressive decline in the number of cell colonies, are shown in Figure 5.

### 3.2. The Cytotoxic Efficiency of Anticancer Agents (Ca^2+^ and BLM) in the Range of US Acoustic Pressures

#### 3.2.1. The Cytotoxic Efficiency of Ca^2+^

**Figure 6:**
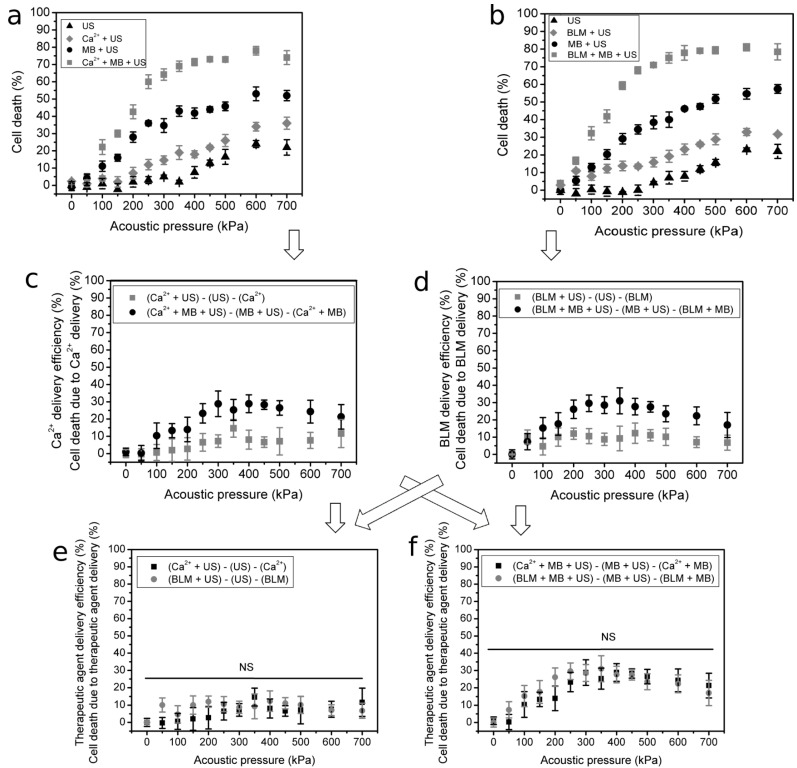
Cell death evaluated using cell clonogenic assay in the range of acoustic pressure for the groups related to Ca^2+^: (US), (Ca^2+^ + US), (MB + US) and (Ca^2+^ + MB + US) (**a**); and BLM: (US), (BLM + US), (MB + US) and (BLM + MB + US) (**b**); the efficiency of Ca^2+^ (**c**) and BLM (**d**) delivery, evaluated as the difference between groups receiving US application or combined MB and US treatment; the comparison of the efficiency of Ca^2+^ and BLM transfer, evaluated after cell exposure to US (**e**) or joint MB and US application (**f**). NS—non-significant.

The predetermined Ca^2+^ concentration of 5 mM was used to evaluate the changes in cell death in the range of acoustic pressures with (Ca^2+^ + MB + US) and without (Ca^2+^ + US) MBs. In addition, important groups of cell death in response to cavitation (MB + US) and US-induced cavitation of the medium (US), were explored. The obtained results (Figure 6a) indicate that US alone (group “US”) starts to evoke cell death at around 300 kPa, but when Ca^2+^ is added to the medium prior to US irradiation (group “Ca^2+^ + US”), cell death is enhanced. Similarly, cell death caused by US-mediated MB cavitation (group “US + MB”) is intensified when the cell medium is supplemented with Ca^2+^ prior to treatment with US (group “Ca^2+^ + MB + US”) (Figure 6a).

With the aim to evaluate the efficiency of Ca^2+^ cytotoxicity, the following subtractions of cell death were performed (Figure 6c):

(i) (Ca^2+^ + US) − (US) − (Ca^2+^) to evaluate the delivery efficiency of Ca^2+^ due to the application of US.

(ii) (Ca^2+^ + MB + US) − (MB + US) − (MB + Ca^2+^) to evaluate the delivery efficiency of Ca^2+^ due to US-induced MB cavitation.

The obtained data (Figure 6c) show that the presence of cavitating MBs increases the efficiency of Ca^2+^ delivery as compared to the application of US.

#### 3.2.2. The Cytotoxic Efficiency of BLM

The standard 20 nM concentration of BLM [7,8,33,34] was used to evaluate changes in cell death in the range of acoustic pressures with (BLM + MB + US) and without (BLM + US) MBs (Figure 6b). The results of cell death within the groups exposed to MB cavitation (MB + US) as well as the application of US alone (US) are presented again since they are related to the corresponding (BLM + MB + US) and (BLM + US) groups and are required for subsequent subtraction.

Similarly, the findings (Figure 6b) indicate that US alone (group “US”) starts to diminish the viability of cells at around 300 kPa, but when BLM is added to the medium prior to US treatment (group “BLM + US”), cell death is increased. The death of cells triggered by cavitation (group “MB + US”) is enhanced when the cell medium is supplemented with BLM prior to cell sonification with US (group “BLM + MB +US”) (Figure 6b).

Similarly, with the aim to evaluate the efficiency of BLM cytotoxicity, the following subtractions of cell death were performed (Figure 6d):

(i) (BLM + US) − (US) − (BLM) to evaluate the delivery efficiency of BLM due to the application of US.

(ii) (BLM + MB + US) − (MB + US) − (MB + Ca^2+^) to evaluate the delivery efficiency of BLM due to US-induced MB cavitation.

The obtained data (Figure 6d) indicate that the presence of cavitating MBs increases the efficiency of BLM delivery as compared to the application of US.

#### 3.2.3. The Efficiency of Intracellular Ca^2+^ and BLM Delivery

The results shown in Figure 6c,d were used to compare the cytotoxic efficiency of both Ca^2+^ and BLM after treatment with US alone (Figure 6e), as well as combined MB and US treatment (Figure 6f). As indicated by the Mann–Whitney test, there is no significant difference between the efficiency of Ca^2+^ and BLM delivery using US (Figure 6e). Similarly, there is no difference in the delivery efficiency of Ca^2+^ and BLM in the presence of MBs and US (Figure 6f).

As indicated by the double titles on the *y*-axes, either Ca^2+^ or BLM delivery efficiency directly reflects the percentage of cells that died due to the intracellular delivery of cytotoxic agents, namely, Ca ions or BLM molecules, respectively. Therefore, as there is no difference in cell death induced by US and Ca^2+^ or BLM (Figure 6e) or by MB and US with Ca^2+^ or BLM (Figure 6f), the level of biological effect’s efficiency is similar between the two therapeutic agents. Therefore, we state that a 5 mM concentration of Ca^2+^ can be used as an alternative to 20 nM BLM in order to enhance cell death. The substitution of BLM with the application of Ca^2+^ for anticancer treatment could avoid the negative side effects of BLM, especially those imposed on off-target locations of the organism, if applied under in vivo conditions.

### 3.3. The Dynamics of Cell Death Induced by the Cytotoxicity of Ca^2+^

**Figure 7:**
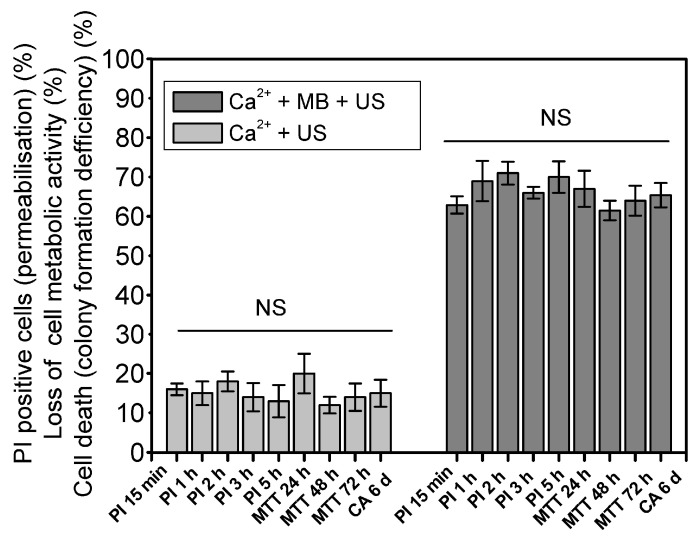
Cell viability dynamics within periods of 15 min–5 h (PI test), 24–72 h (MTT test) or 6 days (clonogenic assay) for (Ca^2+^ + US) and (Ca^2+^ + MB + US) groups at 400 kPa US pressure. Multiple statistical comparisons were performed using ANOVA. NS—non-significant.

The death of CHO cells induced by Ca^2+^ delivery due to US (400 kPa acoustic pressure) exposure with or without MBs was monitored over a 15 min to 6 d period. The results obtained for both (Ca^2+^ + US) and (Ca^2+^ + MB + US) groups were not statistically significant within the same groups (Figure 7).

This result indicates that Ca^2+^ in tandem with SP induces instant cell death, occurring within 15 min, without subsequent cell death from 24–72 h (MTT test) up to a 6 d (clonogenic test) period. This is in contrast to the cell death induced by BLM, which induces cell death after longer time periods—up to 24–48 h—as indicated by the literature [2,32]. This implies fundamentally distinct biological mechanisms for the death of cells induced by Ca^2+^ and BLM after SP. In addition, rapid cell killing, obtained within 15 min after US treatment, may provide anticancer treatment with a fast response, which would be opportune against types of cancer with low doubling times.

### 3.4. The Monitoring of MB Concentration

**Figure 8:**
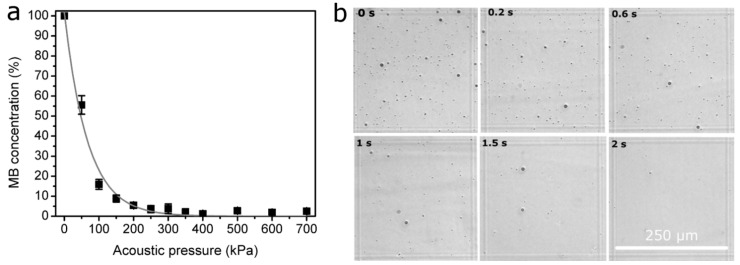
MB concentration evaluated in the range of acoustic pressures after MB exposure to US for a duration of 6 s (**a**); bright-field images of MB suspension, illustrating MB concentration decay within exposure duration at 400 kPa acoustic pressure (**b**).

In order to gain a deeper understanding of the physical mechanism behind Ca^2+^ and BLM delivery via SP, we evaluated the dynamics of the MB concentration after SP. The MB concentration in the range of acoustic pressures after a US exposure of 6 s is presented in Figure 8a.

The results indicate the occurrence of MB sonodestruction at all the acoustic pressures applied, with more than a half of MBs present after irradiation at an acoustic pressure of 50 kPa; in the range of acoustic pressures of 100–300 kPa, only insignificant amounts of MBs remained, whereas total MB sonodestruction occurred in the range of 350–700 kPa. Therefore, it is evident that inertial cavitation occurred in the range of the applied US parameters.

The images produced by the optical microscope (Figure 8b) visually illustrate the gradual decrease in MB concentration within the exposure duration at a constant acoustic pressure of 400 kPa.

### 3.5. The Analysis of Ultrasonic Signals

The analysis of side-scattered US signals generated by cavitating MBs was performed with the aim to gain a deeper understanding of the phenomenon of cavitation, since MB concentration measurements provide information only after the process of SP.

**Figure 9:**
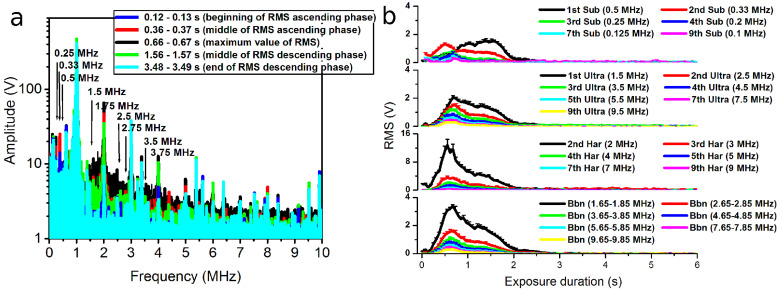
FFT of acoustic pulses recorded at different time intervals of side-scattering signals at 300 kPa acoustic pressure (**a**). The corresponding curves of differential RMS (**b**), quantified in different frequency ranges of the FFT spectra given in (**a**). Arrows in (**a**) indicate the location of harmonic components, which were used for RMS quantification in (**b**). Abbreviations: “Sub”—subharmonic; “Ultra”—ultraharmonic; “Har”—harmonic; and “Bbn”—broadband noise.

The fast Fourier transform (FFT) was applied in order to determine the spectra of frequencies of registered acoustic signals. Figure 9a represents the FFT of acoustic emissions in different frames within a total exposure duration of 6 s at a 300 kPa acoustic pressure. The spectral analysis indicates that acoustic emissions of MBs are present in the whole range of frequencies (up to 10 MHz); however, the major changes occur in the frequency range up to around 4–5 MHz (Figure 9a).

The corresponding differential RMS curves (Figure 9b), quantified in specific frequency regions corresponding to subharmonics, ultraharmonics, harmonics and broadband noise, indicate a gradual decrease in the intensity of cavitating MBs with increasing frequency. This tendency stands as the criterion for the selection of frequency ranges for RMS and CD calculations at the beginning of the frequency spectrum (up to around 4 MHz).

**Figure 10:**
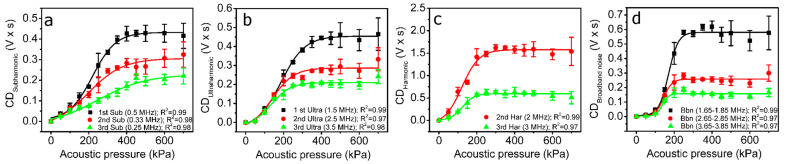
CD, evaluated at different acoustic pressures for subharmonics—CD_Subharmonic_ (**a**); ultraharmonics—CD_Ultraharmonic_ (**b**); harmonics—CD_Harmonic_ (**c**); and broadband noise—CD_Broadband noise_ (**d**). Abbreviations: “Sub”—subharmonic; “Ultra”—ultraharmonic; “Har”—harmonic; and “Bbn”—broadband noise.

Therefore, the frequency spectra of side-scattering signals were used for RMS and, subsequently, CD calculations in different frequency ranges corresponding to ±0.1 MHz around (i) subharmonics, 0.5, 0.33 and 0.25 MHz; (ii) ultraharmonics, 1.5, 2.5 and 3.5 MHz; (iii) harmonics, 2 and 3 MHz; (iv) broadband noise, evaluated within 1.65–1.85, 2.65–2.85 and 3.65–3.85 MHz frequency ranges, which are located between ultraharmonics and harmonics. The resultant CD values for CD_Subharmonic_, CD_Ultraharmonic_, CD_Harmonic_ and CD_Broadband noise_ are presented in Figure 10.

CDs, independently quantified for subharmonics (CD_Subharmonic_), ultraharmonics (CD_Ultraharmonic_), harmonics (CD_Harmonic_) and broadband noise (CD_Broadband noise_), have a sigmoidal shape typical of a dose–response curve and, overall, enter the saturation phase at around 300 kPa.

### 3.6. The Correlation between CD and the Efficiency of Anticancer Agent Delivery

**Figure 11:**
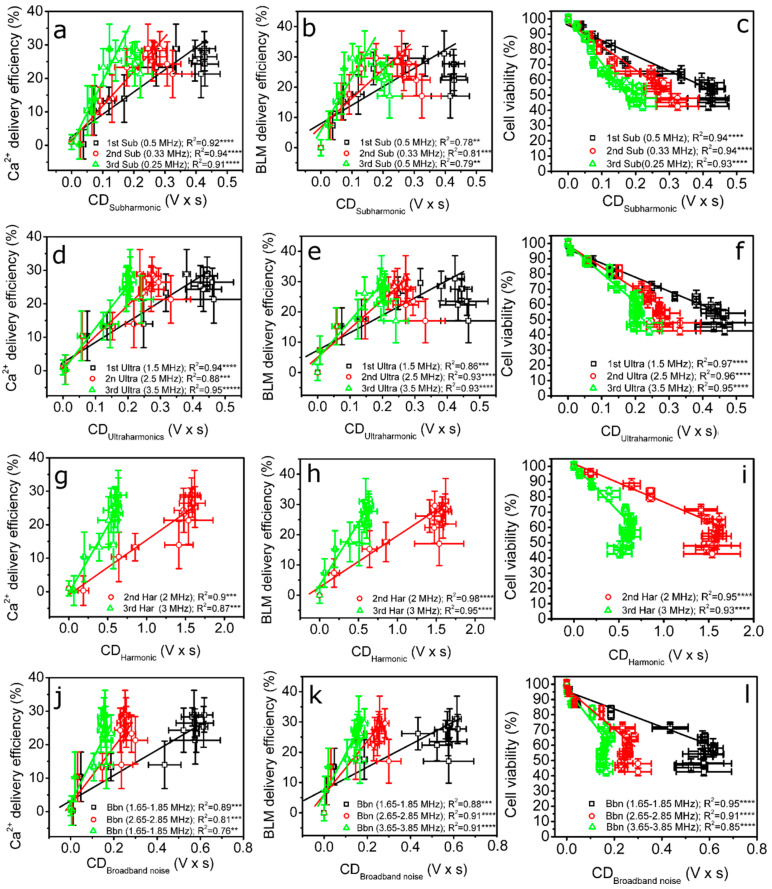
The results of the correlation analysis between CD and the level of the efficiency of the biological effect: CD_Subharmonic_ and the efficiency of Ca^2+^ (**a**) or BLM (**b**) delivery as well as the viability of cells (**c**); CD_Ultraharmonic_ and the efficiency of Ca^2+^ (**d**) or BLM (**e**) delivery as well as the viability of cells (**f**); CD_Harmonic_ and the efficiency of Ca^2+^ (**g**) or BLM (**h**) delivery as well as the viability of cells (**i**); CD_Broadband noise_ and the efficiency of Ca^2+^ (**j**) or BLM (**k**) delivery as well as the viability of cells (**l**). Abbreviations: “Sub”—subharmonic; “Ultra”—ultraharmonic; “Har”—harmonic; and “Bbn”—broadband noise. ** *p* < 0.01; *** *p* < 0.001; **** *p* < 0.0001; ***** *p* < 0.00001.

With the aim to determine the most suitable frequency region for cavitation dosimetry, we performed a detailed correlation analysis between CDs and Ca^2+^ or BLM sonotransfer efficiency, as well as the cell viability results, obtained during SP experiments. The resultant correlation results between CD_Subharmonic_, CD_Ultraharmonic_, CD_Harmonic_ and CD_Broadband noise_ and the efficiency of intracellular Ca^2+^ or BLM delivery, as well as the viability of CHO cells, are presented in Figure 11. The analysis of correlations was performed up to 400 kPa, since the overall delivery efficiency of the anticancer agents increased in this range, whereas, at higher acoustic pressures, it was interfered with by increasing cell death. Therefore, the SP efficiency—evaluated as the difference calculated as (Ca^2+^ + MB + US) – (MB + US) – (Ca^2+^ + MB) or (BLM + MB + US) – (MB + US) – (BLM + MB)—started to decrease.

A correlation analysis was performed for 33 pairs of CD and the efficiency of the biological effect: 22 pairs of correlations of CD and the delivery efficiency of the anticancer agent (Ca^2+^ or BLM) and 11 pairs of correlations of CD and cell viability. In general, the correlation was estimated to be high (R^2^ ≥ 0.8), and only for a few cases did R^2^ drop slightly below 0.8. This indicates that the levels of anticancer agent delivery and cell viability can be well prognosticated using CD, quantified in a broad range of frequencies. Therefore, all the regions of the frequency are informative and can be exploited for cavitation dosimetry. The highest correlation was obtained for ultraharmonics (Figure 11d–f).

## 4. Discussion

In the current research, we performed a detailed evaluation of the cytotoxic efficiency of both Ca^2+^ and BLM in Chinese hamster ovary cells after US- or US-MB-enhanced treatment. The evaluation of the level of the efficiency of biological effect was performed in tandem with the registration of US side-scattered signals using a passive cavitation detection system. The signal analysis aimed to provide valuable information on cavitation dosimetry.

SP is generally considered to be well suited for the intracellular delivery of different anticancer agents. Currently, some of the most often exploited cytotoxic drugs for the treatment of pernicious tumors are BLM and DOX. These agents impose a variety of deleterious secondary effects, such as lung toxicity, gastrointestinal toxicity, hematotoxicity, cardiotoxicity, nephrotoxicity, etc. [12,13]. If these conventional therapeutics are administered by intravascular injection or orally, the damage to healthy tissues and organs is considerably augmented. Therefore, the application of Ca^2+^ is encouraging, since supra-physiological Ca^2+^ concentrations do not cause systemic toxicity to the organism and can be rapidly counteracted by the general regulation of homeostasis and cell-intrinsic mechanisms of Ca^2+^ disposition.

In this study, we found no significant differences between the levels of cell death induced by a 5 mM concentration of Ca^2+^ as compared to 20 nM BLM, delivered using either US alone or US employed in combination with MBs. Therefore, the results indicate that Ca^2+^, at a 5 mM concentration, may be used as an alternative to a traditional chemotherapeutic—BLM. Indeed, Falk et al. showed that Ca^2+^ in combination with US was able to suppress the growth of CT26 colon tumors [35]. This effect was achieved without the employment of MBs; therefore, the therapeutic outcome has the potential to be increased/prolonged.

Relevant studies in the field of EP have reported that the intracellular transfer of Ca^2+^ was able to enhance the death of cancer cells in vitro [36,37] and also reduce tumor growth in vivo [35,37]. Therefore, Ca^2+^ EP has been transferred to clinical practice for the treatment of breast cancer and melanoma [38]. The pioneering study reported no significant difference between the objective response of the patients who received electro-chemotherapy and the response of those treated with Ca^2+^ EP, with fewer side effects reported for the recipients of Ca^2+^. Therefore, the delivery of Ca^2+^ via physical methods may become an encouraging alternative to conventional chemotherapy.

**Figure 12:**
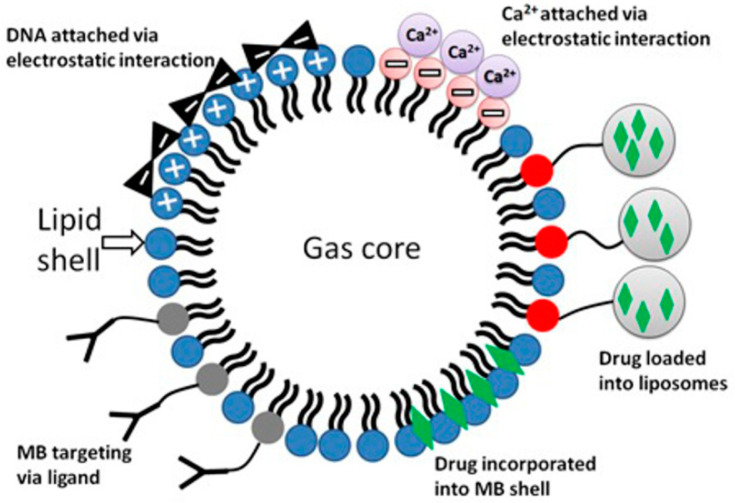
Possible (currently existing) MB modifications for the loading of different therapeutic agents and newly proposed hypothetical modification by incorporating negatively charged lipids into the MB shell to produce anionic MBs, specifically designed for the loading of Ca^2+^.

It was explicitly shown by Lentacker et al. that the anticancer drug DOX can be loaded onto MBs and effectively delivered to cells [5]. Presumably, the employment of MBs as carriers—biological vectors bearing modified negatively charged lipids in the shell and capable of binding Ca^2+^ via electrostatic interaction—is exceptionally promising. The latter would increase the delivery of the payload and ensure efficient antitumor therapy accompanied by enhanced site-specificity and minimal off-target delivery, as well as reduced systemic toxicity. Such a hypothetical Ca^2+^-loaded MB is presented in Figure 12.

The results of cell viability, evaluated with different tests after cell SP, indicate that Ca^2+^ enhances the permeabilization of the cell membrane quickly—within 15 min (PI assay)—with additional changes not observed within either a 24–72 h (MTT test) or 6 d (clonogenic assay) period after treatment. As is extensively described in the literature, the time required for cytotoxic drugs to achieve the effective killing of cells is much higher—up to 24–48 h after SP [2,39,40,41]. By employing four different cell viability tests performed at different time periods after cell EP, our group has shown that cell death, evoked by BLM, was induced 36–48 h after EP [32]. These underlying differences imply different cell death mechanisms behind Ca^2+^ and BLM delivery. In addition, we obtained the same results with different cell viability tests based on different characteristics of cell vitality: the PI assay—the integrity of the cell membrane; the MTT test—metabolic functions of the cell; and the cell clonogenic assay—cell proliferation. This implies that Ca^2+^ delivery via SP significantly impairs all fundamental bases of cell viability and is relevant for the treatment of cancerous diseases. The mechanism of the rapid death of cells after intracellular Ca^2+^ delivery may be similar to that determined in the field of Ca^2+^ EP. Similar to our findings, the rapid death of cells after Ca^2+^ delivery via EP was reported by Frandsen et al. [37]. Cell death was identified as occurring due to cell necrosis, and the mechanism of initiation was associated with Ca^2+^-triggered ATP depletion from cells.

Hoejholt et al. demonstrated that the efficiency of Ca^2+^ or BLM electrotransfer to colon cancer (HT29) and fibroblast (HDF-n) cells is highly dependent on the temperature [36]. Therefore, we have reported the experimental temperature in the methodology section. The temperature plays a significant role, since the intrinsic physicochemical features of the cell membrane are directly altered and affect the level of permeabilization [36]. Thus, the temperature has to be considered as a factor when comparing the results of Ca^2+^ or BLM delivery between different studies.

Since the beginning of the research on cavitation dosimetry, the most suitable frequency range for the calculation of RMS has been discussed. In general, two criteria have been proposed: (i) the quantification of RMS in the frequency range overlapping with the center frequency of the receiver [26]; (ii) the evaluation of RMS at high frequencies (e.g., 9.5–10.5 MHz), since they are more characteristic of broadband noise and inertial cavitation than lower frequencies [42]. However, in order to provide a more general criterion associated with particular types of MBs, we quantified RMS in an extensive range of frequencies associated with subharmonics, ultraharmonics, harmonics and broadband noise (Figure 9b). The results show that RMS undergoes a very homogeneous and gradual increase with decreasing frequency for ultraharmonics and broadband noise; the highest values of RMS were obtained around the first ultraharmonic (1.5 MHz) and broadband noise at 1.65–1.85 MHz, although the receiving transducer had a center frequency of 5 MHz (2.1–7.9 MHz at −6 dB bandwidth). Therefore, these regions could be the most appropriate selection for cavitation dosimetry. Not surprisingly, the 1.5–1.8 MHz frequency range has been identified to be the resonant frequency range for Sonovue MBs [43,44]; therefore, we suggest that the region for cavitation dosimetry be selected within the range of the resonant frequency of MBs. This criterion is associated not with external US equipment (transducers, etc.) but with the type of MBs, since their features are intrinsic to the description of the events of cavitation.

The retention of cavitating nuclei in the regime of stable cavitation is very reasonable because oscillating MBs permeabilize the membranes of nearby cells without collapsing and induce no collateral damage to cells. The frequency spectrum of stably vibrating cavitation nuclei has been identified to bear pronounced subharmonic, ultraharmonic and harmonic components [28,29]. Therefore, quantified CD_Subaharmonic_, CD_Ultraharmonic_ and CD_Harmonic_ in the respective frequencies seem to be correspondingly related to the presence of stable cavitation. The analysis of correlations for most of the pairs identified high (R^2^ ≥ 0.8) and significant (*p* < 0.01) interdependences between CD_Subaharmonic_, CD_Ultraharmonic_ and CD_Harmonic_ and the efficiency of intracellular Ca^2+^ or BLM delivery, as well as the viability of CHO cells. This implies the involvement of stable cavitation in US-MB-mediated therapeutic agent delivery, whereas CD_Broadband noise_ is an estimate of MB inertial cavitation, since collapsing cavitation nuclei generate a frequency spectrum possessing constituents of frequencies between harmonic components [28,29,45]. Therefore, a high (R^2^ ≥ 0.8) and significant correlation between CD_Broadband noise_ and the efficiency of Ca^2+^ or BLM sonotransfer, as well as the viability of cells, denotes the involvement of inertial cavitation in the mechanism of intracellular Ca^2+^ and BLM transfer. Thus, we may assume that both types of cavitation are responsible for the intracellular transfer of anticancer agents. On the other hand, increasing MB cavitation activity (due to an increase in acoustic pressure) evokes an increase in the amplitude of frequency components throughout the whole spectrum of the investigated frequencies (0–10 MHz), as shown in Figure 9b. Therefore, the specific contribution of stable cavitation could be masked by the predominance of inertial cavitation and, presumably, unable to be quantitatively determined using our approach. In this case, a detailed analysis should be performed at lower values of US pressure (up to ~100 kPa); unfortunately, no reliable correlation could be obtained, since the reasonable level of biological effect is initiated only at relatively high acoustic pressures—starting from ~200 kPa (Figure 6f). Therefore, we can only assume that stable cavitation was occurring, as we observed an increase in subharmonic, ultraharmonic and harmonic components in the frequency spectra (Figure 3a and Figure 9a). On the other hand, inertial cavitation (decrease in MB concentration) was also evident, as indicated by total MB sonodestruction at higher acoustic pressures (≥150 kPa) (Figure 8a), as well as the occurrence of broadband noise (Figure 3a and Figure 9a). However, the actual contribution of each mode of cavitation can be argued.

With the aim to avoid additional cell death due to over-threshold inertial cavitation activities and scale down the disparity in experimental findings among different research groups, the development of a standardized measure for cavitation-related bioeffects is necessary. CD satisfies these demands, as it is based on the monitoring of the output characteristics of the system, thereby incorporating and reflecting the variety of biophysical input parameters and milieu of the intrinsic processes taking part in SP. However, CD must be quantified using unified signal processing and metric quantification algorithms in order to associate specific CD values with a particular level of the efficiency of the biological effect among different research groups. This would contribute to the eventual establishment of standardized protocols for in vivo applications and help to bring defined SP-mediated anticancer therapy to the clinical stage.

Our results show that CD has strong correlation-based relations with both the efficiency of the intracellular transfer of Ca^2+^ and BLM as well as the viability of CHO cells. The correlation curves have sparse data for small values at low CD due to the fast increase in Ca^2+^ or BLM sonotransfer efficiency at increasing acoustic pressure (Figure 6f). For the same reason, there are a large number of data points at higher CD values, associated with a higher efficiency of sonotransfer. This tendency implies that MB inertial cavitation is required in order to produce an efficient biological effect, as reported previously [7,8,11,46], and, again, signifies the necessity of SP dosimetry. In addition, we observe a large spread of the higher values of cell viability (Figure 11c,f,i,l) occurring due to a faster saturation of CD values (Figure 10) compared to the saturation of cavitation-induced cell death (group “MB + US” in Figure 6a,b). The same holds for the efficiency of sonotransfer (Figure 11a,b,d,e,g,h,j,k); however, the efficiency of sonotransfer has a slightly larger variation because cell death in the cavitation group (MB + US) saturates at a higher acoustic pressure than in (Ca^2+^ + MB + US) and (BLM + MB + US) groups (Figure 6a,b). Therefore, the efficiency of the sonotransfer starts to decrease at ~400 kPa (Figure 6f). The early saturation of CD curves (Figure 10) might be associated with the lack of recording capacity (frames/s) of the oscilloscope, since RMS curves at high acoustic pressures become short in duration but high in amplitude (Figure 3b). Therefore, the correlation analysis was performed up to 400 kPa, since we are certain of the CD values.

The efficacy of different biological effects can be well prognosticated by CD; therefore, it explicitly indicates that the effectiveness of this particular biological effect can be closely associated with concrete CD values if supplemented with extensive statistical data from the correlation analysis. Indeed, we have previously shown that CD, quantified in 1.65–1.85 MHz, was able to prognosticate the rate of animal survival during the treatment of hepatoma tumors in mouse models [47]. The extensive correlation analysis (33 pairs in total) (Figure 11) shows that CD, quantified in different ranges of frequencies (ranging up to 4 MHz), provides valuable information on the events of cavitation and is suitable for dosimetry. Therefore, each frequency range can be exploited by researchers according to their particular needs; e.g., the selection of the lower-frequency region (1.65–1.85 MHz) is beneficial for in vivo research, since US at low frequencies propagates with lower attenuation in live tissue, implying precise cavitation dosimetry and the control of the treatment [47]. Therefore, the accurate control of cavitation may ensure the safety aspect of sonotherapy with eventual prognostication of the biological outcome.

## 5. Conclusions

1. Ca^2+^ at a concentration of 5 mM can be alternatively used as a substitute for a 20 nM concentration of bleomycin during SP, since they induce the same level of cytotoxicity in Chinese hamster ovary cells.
2. Ca^2+^ delivery via US-MB-assisted treatment elicits a rapid decrease in cell viability, starting within 15 min after US exposure, which remains at a constant level over a 5 h (propidium iodide assay), 24–72 h (MTT test) or 6 d (cell clonogenic assay) period after treatment.
3. The cavitation dose, quantified separately for subharmonics, ultraharmonics, harmonics and broadband noise (up to 4 MHz), was suitable for the prognostication of the cytotoxic efficiency of both anticancer agents, namely, Ca^2+^ and BLM, as was indicated by an overall high (R^2^ ≥ 0.8) correlation (22 pairs in total). The presented extensive statistical data imply that a broad range of frequencies are applicable for the feedback-loop control of the process of US-mediated Ca^2+^ or BLM delivery and can lead to the standardization of protocols for sonoporation.

## Data Availability

The datasets generated during and/or analyzed during the current study are available from the corresponding author on reasonable request.
